# Crude Oil Price Fluctuation Analysis Under Considering Emergency and Network Search Data

**DOI:** 10.1002/gch2.202000051

**Published:** 2020-10-08

**Authors:** Wan‐qiang Dai, Wei Pan, Yongdong Shi, Cheng Hu, Wulin Pan, Ge Huang

**Affiliations:** ^1^ School of Economic and Management Wuhan University Wuhan 430072 China; ^2^ School of Business Macau University of Science and Technology Taipa 999078 China; ^3^ School of Applied Economics Renmin University of China Beijing 100872 China

**Keywords:** case analysis, crude oil price fluctuations, emergencies, network search data

## Abstract

With the rapid development of the global economy, crude oil is becoming more and more prominent in terms of national stability. However, oil prices dramatically fluctuate during emergencies. Meanwhile, network search data have been widely used for prediction during the era of big data. Herein, a suggestion is introduced for improving the traditional case analysis. An autoregressive distributed lag model is established, considering emergency and network search data. Moreover, a network attention index of specific emergencies is used to explain fluctuations of the oil price and the influence of this attention is analyzed. Results show: 1) major emergencies have a significant short‐term impact on the international oil market and a remarkable influence on the cumulative abnormal return of an event window, and 2) market attention can aggravate fluctuations of oil prices. It is found that the individual network attention paid to each of four emergencies has a significant impact on oil prices. The network attention related to Hurricane Katrina and the Libyan war has positive effects on oil prices. However, the effects of network attention paid to the subprime crisis and the Mexico oil spill of 2010 are negative. The attention paid to the subprime crisis has both the greatest and the longest lasting impact.

## Graphical abstract

This paper focuses on the fluctuation of the crude oil price by case analysis considering emergencies. It studies the impact of some typical emergencies on crude oil prices during the reported time window. It also considers the influence of network attention on the crude oil price and calculates cumulative excess return rate during different emergency time windows.

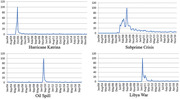

## 1 Introduction

Oil, which is an essential strategic energy source in modern social and economic development, is affected by the world's frequent emergencies, which result in oil prices undergoing severe fluctuations. Taking west texas intermediate (WTI) crude oil spot prices as an example: international oil prices were relatively stable before 2000, with prices remaining at ≈$20 a barrel from 1987 to 1999. Since 2000, the international oil prices have fluctuated more: they increased hugely from 2000 to 2008, rising from ≈$20 a barrel to reach the highest point of $133.88 a barrel in June 2008. However, in February 2009, they fell to $39.09 a barrel, at their lowest point. From 2010 to 2014, oil prices exhibited oscillatory fluctuations, and they then fell dramatically, from $105.79 a barrel in June 2014 to $30.32 a barrel in February 2016. Subsequently, they rose slightly, reaching $51.94 a barrel in August 2017.

Generally, international oil prices fluctuate for a long time, and become more dramatic. Global emergencies are important factors that affect the stability of the oil market. Abnormal fluctuations of oil prices have an essential impact on world economic trends, and these price fluctuations destabilize investment income, bringing risk to producers and consumers alike.

The event study method is widely used to understand the phenomenon of oil price fluctuations caused by emergencies. However, this method cannot determine the exact effect of an emergency in terms of the levels of oil prices. This is mainly because the time window can only be determined subjectively after the incident, and the actual influence of the emergency on the oil price cannot be clarified. Therefore, this paper takes network search data into the analysis framework to improve traditional case analysis. According to the latest report from CNNIC, as of December 2016, China had 731 million Internet users—equal to the total population of Europe—the country's Internet penetration rate had reached 53.2%, and there were more than 600 million users of search engines. Search engines record the user's search behavior, which reflects the attention paid by the user to emergencies, thereby offering both a new perspective and a new data base for market economy behavior analysis.

## 2 Literature Review

Kilian^[^ ^1^ ^]^ simulated and analyzed different types of emergencies, on the assumption that different types of emergencies have different levels of impact on the oil price. Wang et al.^[^ ^2^ ^]^ constructed a transfer function model to both investigate the effect of different emergencies on oil prices and analyze the characteristics of regularity. Coleman^[^ ^3^ ^]^ used a virtual variable to study the effect of emergencies, including the Asian financial crisis, the Kuwait invasion, and the oil worker strike in Venezuela, on oil prices. The authors found that significant emergencies lead to significant fluctuations in oil prices. Chai et al.^[^ ^4^ ^]^ concluded that sudden emergencies could break the oil price equilibrium model, causing great fluctuations in the oil price. Ji and Guo^[^ ^5^ ^]^ used Internet attention to study the impact of four types of related emergencies on oil prices, providing a new perspective for emergency analysis. Based on event analysis, Liao et al.^[^ ^6^ ^]^ studied the effect that incidents and the strategic release of petroleum reserves have on oil prices. They found that the influence of natural disasters is less than that of social conflict and that strategic release of petroleum reserves can help ease prices. Monge et al.^[^ ^7^ ^]^ argued that oil price behavior depends on the emergencies that may disrupt oil transportation, and, through using WTI oil prices to analyze the oil price difference between post‐World War II military conflicts and geopolitical emergencies before and after the war, they found that the oil prices have characteristics of both continuity and interruption. Bompard et al.^[^ ^8^ ^]^ used an overarching methodology to evaluate energy security and applied the methodology to the case of Italy, considering different geopolitical scenarios.

Ginsberg et al.^[^ ^9^ ^]^ proposed a method for tracking influenza‐like cases in people by analyzing a large amount of Google search data. When forecasting tourism product sales in the United States (U.S.). Choi and Varian^[^ ^10^ ^]^ used some keywords’ data as explanatory variables; these new data reference is shown for improving the precision of prediction. Lynn and Erik^[^ ^11^ ^]^ studied the U.S. real estate market: network search data about the real estate market can reflect real estate sales and prices. Lian^[^ ^12^ ^]^ used a web search of China's film market to predict box office data; their results show that an Internet search can predict the rise and fall of movie box office numbers. Li^[^ ^13^ ^]^ used a Baidu index to extract keyword data, forecast the number of the country's tourist attractions tourism; their study shows that network search data can be a good characterization of customer concern. Choi and Varian^[^ ^14^ ^]^ used Google trends data to forecast the unemployment rate in the U.S., finding that a regression forecasting model improves the accuracy when using Google search data. Chong et al. ^[^ ^15^ ^]^ found that there is a co‐integration relationship between network search data and CPI, which has a strong timeliness compared with the traditional prediction model. Li^[^ ^16^ ^]^ analyzed the present situation of big data and its application to oil companies and pointed out that the application of big data to the oil industry is an inevitable trend. Guo and Ji^[^ ^17^ ^]^ used the Internet search patterns of Internet users to study the relationships between oil price fluctuations. Research shows that online attention can reflect the extent of the impact of external emergencies on oil prices. Li et al.^[^ ^18^ ^]^ studied the effect of Google search data on traders’ positions and crude oil prices and suggested that the application of Internet data to oil price analysis and prediction can improve the accuracy of forecasting. Afkhami et al. (2017)^[^ ^19^ ^]^ confirmed the utility of Google search activity by showing that energy‐related keywords are significant predictors of volatility and have incremental predictive power beyond the conventional GARCH models in predicting the volatility of the prices of energy commodities. Campos et al.^[^ ^20^ ^]^ used Heterogeneous Autoregressive (HAR) models that included abnormal search volume from Google (ASVI) and found that this variable has a significant and positive relationship with oil volatility.

All the above studies show that emergencies have significant impacts on oil prices and that different types of emergencies have different effects. network search data can be more accurate in forecast such effects and analysis, related research has risen. Therefore, it is particularly necessary to study the impact of emergency network search data on oil prices.

## 3 Experimental Section

### 3.1 Structural Breakpoint Tests

Breakpoint tests are done to divide the time trend and determine the time of structural change. At present the most sophisticated BP test is a multi‐breakpoint structure test put forward by Bai and Perron.^[^ ^21^ ^]^ According to the program detect the time series of all possible breakpoints directly, it can be obtained by the command of breakdates according to Strucchange software package of R statistical software platform.

**Figure 1:**
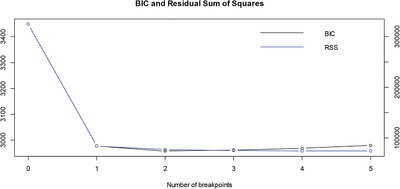
RSS and BIC.

The oil price data in this paper were based on monthly frequency data of WTI crude oil, from May 1987 to December 2016. It can be concluded as shown in **Table** **1** through BP structural breakpoints test, rich site summary (RSS) and bank identifier code (BIC) are shown in **Figure** **1**.

**Table 1:** Breakpoints test

The number of breakpoints	The time of breakpoints					RSS standards	BIC standards
0						324416	3448
1			2005/2			83691	2978
2		1999/11		2005/5		76739	2958
3			2003/1	2007/6	2012/7	74956	2962
4		1998/8	2003/1	2007/6	2012/7	73980	2969
5	1993/5	1998/8	2003/1	2007/6	2012/7	73899	2980

**Figure 2:**
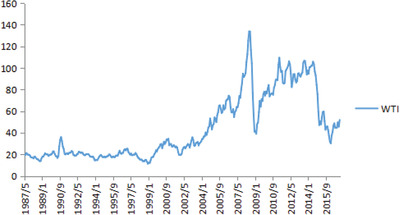
WTI structural breakpoints line mark.

Based on minimum BIC criterion, the optimal number of breakpoints and the breakpoint times were determined; the findings showed that the optimum breakpoint number was 2 and the breakpoint times were November 1999 and May 2005; thus, from May 1987 to December 2016, WTI crude oil spot price movements can be divided into three periods, as shown in **Figure** **2**.

Through the above analysis, oil price fluctuations could be divided into three periods: The first period (May 1987–October 1999) was the stable oil price period; although there were sharp price rises during this time, as there were many wars, oil prices returned to normal rapidly but after emergencies. The second period (November 1999–April 2005) was the rising oil price period; during this period, OPEC cut production three times in a row, causing the international crude oil prices to start rising, and there were several incidents that disrupted oil supplies. The third period (2005–December 2016) was characterized by sudden and sharp rises in oil prices and frequent emergencies, including Hurricane Katrina in August 2005, the subprime crisis in the U.S. in 2008, Iran sanctions, the Libyan war, and the Ukrainian crisis. The analysis shows that occurrence of oil emergencies in the world has become an important factor affecting the stability of oil markets.

### 3.2 Event Study Method

Dolly first proposed the event study method in 1933.^[^ ^22^ ^]^ The method is mainly used to explore the impact of emergencies on price, which is widely used in financial measurement. Using the event study method based on network search data, this paper mainly followed the following steps:

1) Identify the emergency and determine the time window. In the event study method, first specific emergencies should be identified to determine the time window of the emergency. This paper improved the method, using network search data to identify the specific time range of emergencies; set a period when search volume increases and then back to the normal time for the event window. The estimation window was not affected by the event; in this paper, a window range of 140 days was taken before and after the event.
2) Calculate the normal rate of return ER; this paper calculated it using the average rate of return.
3) Calculate abnormal return rate (AR) and cumulative abnormal return rate (CAR)
  (1) $$\begin{array}{c} \begin{array}{c} R_{t}=100\times \ln\left(\frac{P_{t}}{P_{t-1}}\right)\\ \;\;\;\;\;\;\;{\text{AR}}_{t}=R_{t}-{\text{ER}}_{t}\\ \;\;\;\;\text{CAR}=\sum {\text{AR}}_{t} \end{array} \end{array}$$
4) By testing CAR and ER, the significant level of events was determined. In accordance with previous studies, it is assumed that the abnormal return rate obeys the Gaussian distribution and the mean is zero; it is also assumed that it is independent identically distributed
  (2) $$\begin{array}{c} \theta \;=\frac{\text{CAR}}{\sqrt{\mathrm{var}\left(\text{CAR}\right)}}\;∼N\left(0,1\right) \end{array}$$

**Figure 3:**
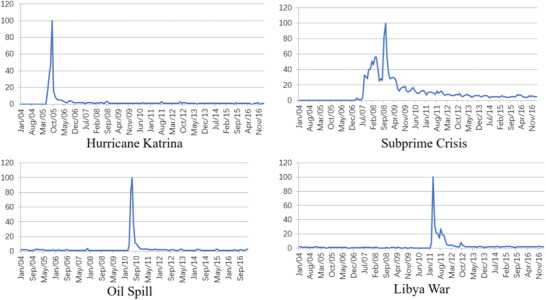
Emergency network attention.

Based on the above analysis, this paper selected different types of relatively typical emergencies to analyze: these were Hurricane Katrina (natural disaster) in 2005, the subprime crisis (economic crisis) in 2008, the Mexico oil spill in 2010 (disaster), and the Libyan war (conflict) in 2011. The Brent oil spot price was chosen to describe international oil prices; data come from the U.S. Energy Information Administration (EIA).^[^ ^23^ ^]^ Search volume data provided by Google trends were selected for analysis; the search volume was relative data and the number of times the input word was searched, which was relative to the total Google searches in a period of time in the past; thus the data reflected the attention of particular keywords during a certain period. Based on the research object of this paper, the initial keywords of emergencies were: Hurricane Katrina, Subprime Crisis, Oil Spill, and Libya war. Their network attention is shown in **Figure** **3**, and the relevant data are shown in **Table** **2**.

**Table 2:** Yield models

	Event window	Yield models
Hurricane Katrina	2005/08/29–09/23	*R_t_* = 0.291 + *e_t_*, *e_t_* ≈ *N* (0,3.89)
Subprime crisis	2005/09/15 –12/31	*R_t_* = 0.1579 + *e_t_*, *e_t_* ≈ *N* (0,1.829)
Oil spill	2010/04/20–07/30	*R_t_* = −0.0228 + *e_t_*, *e_t_* ≈ *N* (0,5.12)
Libya war	2011/02/22–04/01	*R_t_* = 0.1798 + *e_t_*, *e_t_* ≈ *N* (0,3.495)

**Figure 4:**
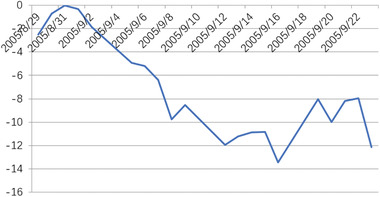
CAR of Hurricane Katrina.

**Figure 5:**
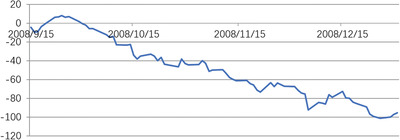
CAR of subprime crisis.

**Figure 6:**
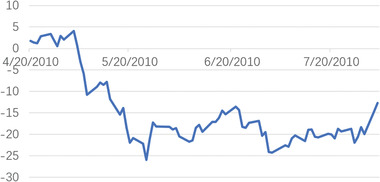
CAR of oil spill.

**Figure 7:**
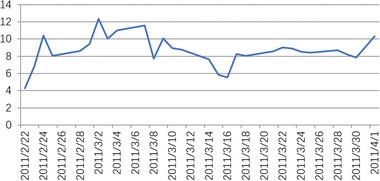
CAR of Libya war.

When heteroscedasticity and autocorrelation tests were carried out on the model, it was found that they did not obey the original assumption. This indicated that the model set was appropriate. Then, the abnormal return AR, and the cumulative abnormal return CAR were calculated. In the event window, the trend of the abnormal rate of return is shown in **Figures** **4**–**7**.

The CAR of Hurricane Katrina in the event window showed an obvious downward trend, only 2 days before the emergency occurred with a modest rise, then there had been negative. According to the test results, Hurricane Katrina had a significant negative effect on the CAR of Brent crude oil. After the incident, the release of the strategic petroleum reserve (SPR) by the U.S. government on August 31 played an important role in easing prices, reducing the price of crude oil price. Overall, the effect of Hurricane Katrina on the crude oil market degree was small and short‐lived.

According to the results of CAR statistics, the subprime crisis has had a significant negative impact on the CAR of Brent crude oil. In general, the subprime crisis had caused a serious setback in market confidence, which had impacted oil demand and has had a long‐lasting and large effect on the oil market.

The CAR change trend showed that, on the day of the occurrence of an emergency, CAR was positive and rose slightly, whereas, in the period after the incident, it fell sharply to a negative value and remained at a low level. During the early stage of the emergency, people overestimated the consequent supply shortage problem and showed an excessive reaction to the emergency; whereas, when the data were announced, the closed platform accounted for only 0.1% of normal daily output and the impact of the oil spill was limited, therefore the market callback, ultimately the representation of the CAR first rose and then fell. From the test results, at the 5% significance level, the Mexico oil spill emergency had significant negative effects on the CAR of the Brent crude oil price, which suggests that, in the short‐term, the Mexico oil spill caused oil prices to rise slightly, although the market overreaction ultimately resulted in a declining trend for oil prices.

From the change trend of CAR, it could be seen that, in the event window, the Libyan war CAR showed a rise at first and then concussion; in the initial stage (3 days before the event window) the CAR rose sharply. According to the test results, the Libyan war had a significant positive influence on the CAR of Brent crude oil, showing that the Libyan war caused oil prices to rise obviously in the short term, but the impact did not last for long.

By summarizing and drawing conclusion from the above research, it can be found that: 1) In general, major emergencies had a significant short‐term impact on the international oil market and contribute to the volatility of oil over a short period of time; 2) The influence of different types of emergencies on the direction and duration of oil prices was different; 3) The empirical results showed that Hurricane Katrina, the subprime crisis, and the Mexico oil spill had a significant negative impact on CAR within the event window, while the Libyan war had a positive effect; 4) At the same time, the mastery degree of market information and the strategy of releasing the SPR could effectively affect the impact of emergencies. It was necessary to contact the particularity of emergencies and environment to analyze the influence situation.

## 4 Econometric Models

The empirical results of these four different types of emergencies show that emergencies within the event window have a significant impact on CAR. For further analysis of the effects of specific incidents on oil price fluctuations, we use network search data provided by Google trends and construct emergency attention indicators, and we use emergency attention to explain oil price fluctuations and measure the size of the specific influence of different types of emergencies.

### 4.1 Data Preprocessing

Through constructing an econometric model and attention indicators based on the network search data, we go on to investigate the effect of emergencies on oil prices.

In this paper, the dependent variable (*Y*) is the price of oil, using Brent crude oil spot price Price*_t_*; the data come from the EIA. According to the time emergencies happen, we make time range from January 4, 2005 to December 30, 2005, from January 2, 2008 to December 31, 2008, from January 4, 2010 to December 31, 2010, from January 3, 2011 to December 30,2011, all 1010 price data during trading days. The independent variable (*X*) of this article is network search data Concern*_t_*, certain keywords related to the emergencies. The search volume is relative data and the number of times the input word is searched, which is relative to the total Google searches in a period of time in the past; thus, the data reflect the attention paid to particular keywords during a certain period. Here, search data only retain trading day data to maintain the oil price data consistency.

In this paper, the steps taken to select and search for keywords are as follows.

First, based on the research object of this paper, we set an initial keyword library related to Hurricane Katrina, the subprime crisis, the Mexico oil spill, and the Libyan war.

Second, according to relevant keywords recommended by Google trends,^[^ ^24^ ^]^ we expand the keyword library based on the initial keywords.

Third, we filter keywords according to the correlation of oil price fluctuation, finding words with a bigger correlation index, and then synthesize them. We compound keywords to synthesize many search keywords that can reflect the change of dependent variable, until we get a unified index and predict corresponding variables. To guarantee the stability of the correlation index, we use time difference correlation analysis to verify the correlation between keyword and oil price fluctuations, and we calculate the correlation coefficient of each keyword eight times, namely using the oil price on the day and each keywords Google search data in advance of 0 to 7 days calculate correlation index respectively. Assuming *Y* = {*Y*_1_, *Y*_2_, *Y*_3_, …, *Y_n_*} is research data, *X* = {*X*_1_, *X*_2_, *X*_3_ … *X_n_*} is indicators to be selected, and *R_t_* is the time difference correlation index, then

(3) $$\begin{array}{c} R_{t}=\frac{\sum_{i=1}^{n}\left(X_{i-t}-\bar{X}\right)\left(Y_{i}-\bar{Y}\right)}{\sqrt{\sum_{i=1}^{n}\left(X_{i-t}-{\bar{X}}^{2}\right)\;\sum_{i=1}^{n}\left(Y_{i}-{\bar{Y}}^{2}\right)}} \end{array}$$

where *t* = ±1, ±2, ±3, …, ±*T*.

The meanings of each variable in the formula are: *R_t_* is the time difference correlation index, *Y* is the everyday oil price, $\bar{Y}$ is the mean of the oil price, *X* is the everyday research data of keywords, $\bar{X}$ is the mean of the research data, and t is the leading order when the *X* order correlation index is the biggest leading time order_∘_

This paper uses keywords whose relation index is bigger than 0.5 to construct an emergencies’ keywords library, as shown in **Table** **3**.

**Table 3:** Keywords of emergency

Emergency	Keywords finally	Number
Hurricane Katrina	Hurricane Katrina, Hurricane Katrina new Orleans, Katrina Hurricane facts, Hurricane Katrina damage, oil production, oilfield shutdown, facts about Hurricane Katrina, Hurricane Katrina statistics	8
Subprime crisis	Subprime crisis, mortgage crisis, subprime mortgage, financial crisis, subprime lending, subprime loans, Lehman Brothers, collapse of Lehman Brothers, facts of 2008 crisis	9
Oil spill	Oil spill, the bp oil spill, gulf of Mexico, 2010 bp oil spill, oil spill in Mexico, oil spill facts	6
Libya war	Libya war, Libya civil war, oil production, military conflict 2011, war on Libya, political conflict 2011, Libya facts	7

Then, using the weighted average method to synthesize keywords, the corresponding weight is the time difference correlation index of keywords and oil price fluctuation. The logarithm of the dependent variable and independent variables above are LPrice*_t_* and LConcern*_t_*.

### 4.2 Constructing Models

To investigate the influence of emergency attention on oil price fluctuations, this paper uses oil price data and emergency network search visibility data as independent variables and incorporates these into the model, taking into account the quality of hysteresis of network search visibility. We establish the model as follows

(4) $$\begin{array}{c} \begin{array}{c} {\text{LPrice}}_{\text{t}}=c+\alpha_{1}{\text{LPrice}}_{\left(t-1\right)}+\alpha_{2}{\text{LPrice}}_{\left(t-2\right)}+\cdots +\alpha_{p}{\text{LPrice}}_{\left(t-p\right)}\\ \;+\beta_{1}{\text{LConcern}}_{\left(t-1\right)}+\beta_{2}{\text{LConcern}}_{\left(t-2\right)}+\cdots +\beta_{p}{\text{LConcern}}_{\left(t-p\right)}+\varepsilon_{t} \end{array} \end{array}$$

where *t* = 1, 2, …, *n*, *p* = 1, 2, …, *n*, *q* = 0, 1, 2, …, *n*.

LPrice*_t_* is the everyday oil price in logarithmic form everyday oil price after being logarithm. So many factors can influence the oil price that it is difficult to incorporate them all in the model. Consequently, we follow Ji and Guo,^[^ ^5^ ^]^ by introducing price data LPrice_(_ *_t_*_−_ *_p_*_)_ which phase lag *p* to measure market factors other than the specific incident. LConcern*_t_* is the everyday network research data after being in logarithmic form, and it was synthesized by a Google search data of emergency keywords. *C* is a constant term, and *ε_t_* is a residual term.

Then LM test is taken for the residual sequence, if autocorrelated, go on ARCH effect testing. If it shows a high order ARCH effect, namely GARCH effects, we establish a generalized autoregressive conditional heteroscedastic model; the final model is as follows

(5) $$\begin{array}{c} \begin{array}{c} {\text{LPrice}}_{\text{t}}=c+\alpha_{1}{\text{LPrice}}_{\left(t-1\right)}+\alpha_{2}{\text{LPrice}}_{\left(t-2\right)}+\cdots +\alpha_{p}{\text{LPrice}}_{\left(t-p\right)}\\ \;+\beta_{1}{\text{LConcern}}_{\left(t-1\right)}+\beta_{2}{\text{LConcern}}_{\left(t-2\right)}+\cdots +\beta_{p}{\text{LConcern}}_{\left(t-p\right)}+\varepsilon_{t} \end{array} \end{array}$$

(6) $$\begin{array}{c} \varepsilon_{t}∼N\left(0,h_{t}\right) \end{array}$$

(7) $$\begin{array}{c} h_{t}=\gamma_{0}+\gamma_{1}\varepsilon_{t-1}^{2}+\gamma_{2}h_{t-1} \end{array}$$

where *t* = 1, 2, …, *n*, *p* = 1, 2, …, *n*, *q* = 0, 1, 2, …, *n*.

Formulas (6) and (7) define conditional variances of LPrice*_t_*.

To ensure that the time series is stable and to avoid a spurious regression, it is necessary to perform a stationarity test for all variables; this paper uses the augmented dickey‐fuller (ADF) test method. If the inspection results reject the null hypothesis, this sequence is smooth, otherwise it is unsmooth, and we continue to take difference tests until it is smooth. The unit root test results of time series data are shown in **Table** **4**. This table shows that all variables are of first‐order difference declined to the original assumption, argues that they are one single integer sequences, namely, I (1) process, achieving the premise condition of cointegration test, then cointegration test is taken, examining whether there is a long‐term equilibrium relationship between the sequences.

**Table 4:** Unit root test results

Emergency	Variables	ADF test	ADF test results
Hurricane Katrina	LPrice_2005_	−1.9253	Nonstationary
	ΔLPrice_2005_	−9.9391^**^	Stationary
	LConcern_2005_	−4.4460^**^	Stationary
	ΔLConcern_2005_	−10.1007^**^	Stationary
Subprime crisis	LPrice_2008_	−0.1063	Nonstationary
	ΔLPrice_2008_	−16.7054^**^	Stationary
	LConcern_2008_	−4.9588^**^	Stationary
	ΔLConcern_2008_	−23.4551^**^	Stationary
Oil spill	LPrice_2010_	−2.4068	Nonstationary
	ΔLPrice_2010_	−12.9627^**^	Stationary
	LConcern_2010_	−5.0764^**^	Stationary
	ΔLConcern_2010_	−11.8371^**^	Stationary
Libya war	LPrice_2011_	−4.1944^**^	Stationary
	ΔLPrice_2011_	−14.3957^**^	Stationary
	LConcern_2011_	−14.3249^**^	Stationary
	ΔLConcern_2011_	−24.8777^**^	Stationary

^***^1%, ^**^5%, ^*^10%.

This paper adopts two steps cointegration relationship tests, and the results are shown in **Table** **5**. As can be seen from these results, based on the ADF test of the residual sequence *e*, the residual sequence *e_t_* is stationary series, there is a long‐term stable cointegration relationship between network search attention paid to emergencies and oil prices.

**Table 5:** Cointegration test results

		Model (1)	Model (2)	Model (3)	Model (4)
Variables		Hurricane Katrina	Subprime crisis	Oil spill	Libya war
*c*		0.5824* (2.6297)	−0.0560* (−2.0172)	0.2779* (2.5545)	0.3942^**^ (3.0043)
LPrice_(_ *_t_*_−1)_		0.6655^**^ (13.5806)	0.9101^**^ (184.9911)	0.7385^**^ (38.5538)	0.8167^**^ (33.1973)
LConcern*_t_*		0.0337^**^ (3.4348)	−0.0578^**^ (−4.5117)	−0.0236^**^ (−3.6078)	0.0524^**^ (4.1627)
LConcern_(_ *_t_*_−1)_			−0.0752^**^ (−4.1724)		
Adjusted *R* ^2^		0.9135	0.9928	0.9472	0.9314
*F*‐value		470.86	11 482.82	1598.43	563.15
Residual stationarity	ADF value	−9.4403^**^	−16.4871^**^	−12.6632^**^	−13.8226^**^
	1%	−3.5056	−3.4563	−3.4672	−3.4612
	5%	−2.8943	−2.8729	−2.8776	−2.8750
	10%	−2.5843	−2.5729	−2.5754	−2.5740
	ADF text results	Stationary	Stationary	Stationary	Stationary
Conclusion		Cointegration	Cointegration	Cointegration	Cointegration

^***^1%, ^**^5%, ^*^10%.

We both model and estimate four different emergencies; according to the estimation results, web search attention related to the four emergencies has a significant impact on oil prices. The adjusting values *R* ^2^ of four models are above 0.9, according to the *F*‐value, models are significantly as a whole.

The coefficient of LConcern*_t_* shows that network search attention to different types of emergencies has different impacts on both direction and price. The coefficients of LConcern*_t_* for Hurricane Katrina and the Libyan war are positive, suggesting that the attention paid to the two incidents aggravated the increases of oil prices. Meanwhile, the same coefficients for the subprime crisis and the oil spill are negative, suggesting that the attention paid to these two incidents caused decreases in oil price. The size of the coefficient of LConcern*_t_* correlates with the degree of influence on the oil price. The largest effect was seen with the attention paid to the subprime crisis. Next, we interpret the four models and analyze the influence specifically.

### 4.3 Results Analysis

Through the econometric model, we operate the data and obtain the results shown in **Table** **6**.

**Table 6:** Network attention comparison

Emergency	Hurricane Katrina (2005)	Subprime crisis (2008)	Oil spill (2010)	Libya war (2011)
Type	Nature disaster	Economic crisis	Event disaster	War conflict
Coefficient	0.0337	−0.0752 (*t*−1) −0.0578 (*t*)	−0.0236	0.0524
Significant	Yes	Yes	Yes	Yes
Direction	Positive	Negative	Negative	Positive

#### 4.3.1 Hurricane Katrina

A Lagrange multiplier (LM) test is applied to the residual sequence of mode (1); the concomitant probability *p* > 0.05, which means that, at a 5% significance level, the residual sequence does not relate. According to model (1), we establish the following equation

(8) $$\begin{array}{c} {\text{LPrice}}_{t}=0.5824+0.6655{\text{LPrice}}_{\left(t-1\right)}+0.0337{\text{LConcern}}_{t} \end{array}$$

The independent variable LPrice_(_ *_t_*_−1)_ is a history term, which is mainly used to describe other factors, except for the effect of the Hurricane Katrina emergency on oil prices; the results of our calculation show that oil prices are significantly correlated at both the *t* stage and the *t* − 1 stage of the oil price, with a coefficient of 0.6655.

LConcern*_t_* is a search term, reflecting changing market attention to Hurricane Katrina. The variable coefficient is 0.0337, which is positive and significant at a 1% significance level, meaning that market awareness of Hurricane Katrina has a significant positive effect on oil prices, and the improvement of search attention can lead to the rising oil prices. Variations in the price of the attention change 1%, corresponding to 0.0337% of oil price. Hurricane Katrina belongs in the category of sudden natural disasters, the duration of which is temporary; such emergencies lead to fluctuations in oil prices in the short term, mainly by increasing the uncertainty of the oil market.

On August 30, the emergency day, Brent crude oil spot prices rose from $64.77 a barrel to $66.15 a barrel, but the U.S. government decided to release SPR on August 31. When the IEA announced the release of SPR on September 2, market confidence grew stronger, and the increase in oil prices was suppressed quickly. Therefore, when the information about the Hurricane Katrina emergency reached the oil market, oil prices rose with increasing attention, and they then gradually fell for the drop of information uncertainty, until oil prices returned to normal levels.

#### 4.3.2 Subprime Crisis

LM test is taken in the residual sequence of model (2); the concomitant probability *p* > 0.05, which means that, at a 5% significance level, the residual sequence is not related. According to model (2), we establish the following equation

(9) $$\begin{array}{c} \begin{array}{l} {\text{LPrice}}_{t}=-0.0560+0.9101{\text{LPrice}}_{\left(t-1\right)}-0.0578{\text{LConcern}}_{t}\\ \;\;\;\;\;\;\;\;\;\;\;\;\;\;\;-0.0752{\text{LConcern}}_{\left(t-1\right)} \end{array} \end{array}$$

The independent variable LPrice_(_ *_t_*_−1)_ is a history term, which is mainly used to describe other factors, except for the effect of the subprime crisis on oil prices; the results of our calculation show that oil prices are significantly correlated at both the *t* stage and the *t* – 1 stage of the oil price, with a relatively large coefficient of 0.9101.

LConcern*_t_* is a search term, reflecting changing market attention to the subprime crisis. LConcern*_t_* is current research data, and it is positive and significant at a 1% significance level, with a coefficient of −0.0578, meaning that market awareness of the subprime crisis has a significant negative effect on oil prices and that increased search attention can lead to oil prices falling. Variations in the price of the attention change 1%, corresponding to −0.0578% of oil price. LConcern_(_ *_t_*_−1)_ represents research data from an earlier period and it has a coefficient of −0.0752, meaning that variations in the price of the attention change 1%, corresponding to −0.0752% of the oil price.

Different from Hurricane Katrina, the impact of awareness of the subprime crisis awareness on oil prices exist the conduction lag, which not only affects the current oil prices but also can produce continuous function. The absolute value of the current search volume regression coefficient is less than is that of the earlier search volume regression coefficient, explain that the influence effect is weakened gradually. Different from sudden natural disasters, the market economic issues of the subprime crisis can be forecast in advance, this also causes a collapse of market confidence, resulting in oil prices plunging, and aggravates the effects of the emergencies. For this kind of emergency, market information plays an important role, with the increasing spread of market information and market awareness and the tumbling of market confidence impacting the international oil demand, leading to the oil supply and demand imbalances and oil prices continuing to fall. The subprime crisis has a considerable and long‐lasting effect on the oil market.

#### 4.3.3 Oil Spill

LM test is taken in the residual sequence of model (3); the concomitant probability *p* > 0.05, which means that, at a 5% significance level, the residual sequence is not related. According to model (3), we establish equation as follows

(10) $$\begin{array}{c} {\text{LPrice}}_{t}=0.2779+0.7385{\text{LPrice}}_{\left(t-1\right)}-0.0236{\text{LConcern}}_{t} \end{array}$$

The independent variable LPrice_(_ *_t_*_−1)_ is a history term, which is mainly used to describe other factors, except for the effect of the oil spill on oil prices. The results of our calculation show that oil prices are significantly correlated at both the *t* stage and the *t* – 1 stage of the oil price, with a coefficient of 0.7385.

LConcern*_t_* is a search term, reflecting the changing market attention to the oil spill. It is positive and significant at a 1% significance level, with a coefficient of −0.0236, meaning that market awareness of the oil spill has a significant negative effect on oil prices, and increased search attention can lead to oil prices falling. Variations in the price of the attention change 1%, corresponding to −0.0236% of the oil price. Different from the subprime crisis, which can be predicted in advance, the oil spill is a sudden disaster that cannot result in a persistent imbalance between supply and demand. The incident initially causes a decline in oil supplies, but the effect is short and mild. Unlike our expectations, the oil spill has not led to the increase of oil prices; on the contrary the increasing market attention to the oil spill has led to lower oil prices. At the beginning of the emergency, people overestimate the potential shortage problem arising from it and overreact to it; with the announcement of actual data, the closed platform accounts for only 0.1% of the normal daily output. The impact of the oil spill is limited, and the market callback, and the oil price finally shows a downward trend. This shows that market information can aggravate the impact of emergencies and can even reverse the effect and increase uncertainty in the oil market.

#### 4.3.4 Libyan War

LM test is taken in the residual sequence of model (4), different from the first three models, the concomitant probability *p* = 0, which means that the residual sequence is related. Go on the ARCH effect test, results are shown in **Table** **7**. When *q* = 8, the concomitant probability *p* = 0.0022, so the residual sequence has a high order ARCH effect, namely the GARCH effect. Therefore, we establish the generalized autoregressive conditional heteroscedastic model.

**Table 7:** ARCH text results

*F*‐value	3.2727	Prob. *F*	0.0016
Adjusted *R* ^2^	24.1573	Prob. Chi‐square	0.0022

Based on Akaike information criterion (AIC) and Schwarz criterion, we establish the GARCH (1,1) model as follows

(11) $$\begin{array}{c} \begin{array}{c} {\text{LPrice}}_{\text{t}}=c+\alpha_{1}{\text{LPrice}}_{\left(t-1\right)}+\alpha_{2}{\text{LPrice}}_{\left(t-2\right)}+\cdots +\alpha_{\text{p}}{\text{LPrice}}_{\left(t-p\right)}\\ \;+\beta_{1}{\text{LConcern}}_{\left(t-1\right)}+\beta_{2}{\text{LConcern}}_{\left(t-2\right)}+\cdots +\beta_{p}{\text{LConcern}}_{\left(t-p\right)}+\varepsilon_{t} \end{array} \end{array}$$

(12) $$\begin{array}{c} \varepsilon_{t}∼N\left(0,h_{t}\right) \end{array}$$

(13) $$\begin{array}{c} h_{t}=\gamma_{0}\;+\gamma_{1}\varepsilon_{t-1}^{2}+\gamma_{2}h_{t-1} \end{array}$$

where *t* = 1, 2, …, *n*, *p* = 1, 2, …, *n*, *q* = 0, 1, 2, …, *n*.

Formulas (12) and (13) define the conditional variance of LPrice*_t_*. Results of the GARCH (1,1) model are shown in **Table** **8**.

**Table 8:** Results of GARCH (1,1) model

Variables	*c*	LPrice_(_ *_t_*_−1)_	LConcern*_t_*	γ_0_	$\varepsilon_{t-1}^{2}$	*h*_t−1_	Adjusted *R* ^2^
Model (5)	0.3942^**^ (53.012)	0.8167^**^ (16.921)	0.0524^**^ (3067.413)	2.39E−06^**^ (14.356)	0.1500^**^ (35.612)	0.6210^**^ (40.896)	0.9426

^***^1%, ^**^5%, ^*^10%.

As can be seen from **Table** **9**, compared with model (4) which adopts the autoregressive distributed lag model, the adjusted *R* ^2^ of model (5) is 0.9426, which is an increase of 1.2% and a better fit, and the AIC and AC values are reduced accordingly, so the model (5) is more appropriate.

**Table 9:** Comparison of models

Model	AIC	SC	Adjusted *R* ^2^
Model (4)	−20.5638	−20.5165	0.9314
Model (5)	−24.3534	−24.2588	0.9426

According to model (5), we establish the following equation for the Libyan war

(14) $$\begin{array}{c} {\text{LPrice}}_{t}=0.3942+0.8167{\text{LPrice}}_{\left(t-1\right)}+0.0524{\text{LConcern}}_{t}+\varepsilon_{t} \end{array}$$

(15) $$\begin{array}{c} \varepsilon_{t}∼N\left(0,h_{t}\right) \end{array}$$

(16) $$\begin{array}{c} H_{t}=2.39\text{E}-06+0,15\varepsilon_{t-1}^{2}+0.62h_{t-1} \end{array}$$

The independent variable LPrice_(_ *_t_*_−1)_ is a history term, which is mainly used to describe other factors, except for the effect of the Libyan war on oil prices; the results of our calculation show that oil prices are significantly correlated in both the *t* stage and the *t* – 1 stage of the oil price, with coefficient of 0.8167.

LConcern*_t_* is a search term, reflecting the change in market attention to the Libyan war. It is positive and significant at a 1% significance level, with a coefficient of 0.0524, meaning that market awareness of the Libyan war has a significant negative effect on oil prices, and increased search attention can lead to higher oil prices. Variations in the price of the attention change 1%, corresponding to 0.0524% of the oil price.

For the variance equation, the coefficient γ_2_ of *h_t_*_−1_ in model (5) is 0.621, indicating that 62.1% of current period impact is still present in the next issue. The impact of the Libyan war on oil price volatility diminishes and eventually reaches zero. The half‐life can be calculated by the value of this coefficient

(17) $$\begin{array}{c} \frac{\log0.5}{\log\gamma_{2}}=1.45 \end{array}$$

According to the results, the half‐life of the Libyan war's impact on the oil market is one to two days, which indicates that the oil price fluctuation caused by this emergency has a relatively short duration. Like Hurricane Katrina, the Libyan war also belongs to the category of supply shocks; such incidents in the oil market supply are mainly short‐lived, causing oil prices to rise in the short‐term; however, with increasing improvement of the oil market environment and the market system mechanism, the impact of such emergencies is increasingly limited. Network search data reflect the market attention and master degree of information, affecting people's psychological expectations and increasing market uncertainty, thus, to some extent, aggravating the effects of emergencies; however, the degree of influence shows a tendency for marginal decline, eventually reducing to zero.

## 5 Conclusion

The above analysis shows that 1) Attention from Internet searches of emergencies has a significant impact on oil prices, with different types of emergencies having different effects and directions. 2) After an incident, supply shock causes the oil market supply in a short‐time, leading to a continuous rise in oil prices. However, with increasing improvement of the oil market environment and the market system mechanism, the impact of such emergencies is increasingly limited. 3) At the same time, information serves as a bridge between investors and oil markets, transmitting emergency information and influencing people's psychological expectations, thereby affecting the oil market. 4) As available quantitative data, the network search data of relevant emergencies both provide a new perspective on and prompt new thinking about the quantitative analysis of oil emergencies.

We have enlightenment as follows: 1) China, as oil demand power, decision‐making processes concerning oil imports should take the characteristics of different incidents into consideration, i.e., oil should be imported when a demand shock occurs, and oil imports should be reduced by as much as possible when a supply shock occurs; 2) In the oil market, the risk of emergencies should not be ignored, and full and accurate market information plays an important role in predicting the oil price trend and reducing the impact of emergencies; 3) In the process of handling major emergencies, release of the SPR can inhibit oil price fluctuations, relieving the shock effect of the incident shock. Therefore, the SPR system is of great significance in ensuring national energy security; 4) Network search data with real‐time characteristics can monitor and track price change trend in real‐time, making up for the lag in statistical data. According to the degree of the attention, network search data provide a reasonable evaluation of the incident and help in the formulation and implementation of related policies and measures in the oil market.

## Conflict of Interest

The authors declare no conflict of interest.

## Author Contributions

W.‐q.D. and W.P. contributed equally to this work as the co‐first authors. All authors were involved in the writing and revision of the manuscript. All authors read and approved the final version.

## References

[1] L. Kilian , Am. Econ. Rev. 2009, 99, 1053.

[2] S.‐p. Wang , Y. Chen , Y. Jin , Math. Pract. Theory 2009, 9, 88.

[3] L. Coleman , Energy Policy 2012, 40, 318.

[4] J. Chai , Z. Zhang , J. Fu , J. Guo , S. Wang , J. Manage. Sci. 2014, 27, 133.

[5] J. Qiang , J.‐F. Guo , Appl. Energy 2015, 137, 256.

[6] S. Liao , F. Wang , T. Wu , W. Pan , Energy 2016, 102, 436.

[7] M. Monge , L. A. Gil‐Alana , F. Pérez de Gracia , Energy 2017, 120, 79.

[8] E. Bompard , A. Carpignano , M. Erriquez , D. Grosso , M. Pession , F. Profumo , Energy 2017, 130, 144.

[9] J. Ginsberg , M. H. Mohebbi , R. S. Patel , L. Brammer , M. S. Smolinski , L. Brilliant , Nature 2009, 457, 1012. 19020500 10.1038/nature07634

[10] H. Choi , H. Varian , Predicting the Present with Google Trends, Working Paper, 2009.

[11] L. Wu , B. Erik , The Future of Prediction—How Google Searches Foreshadow Housing Prices and Sales, Working Paper, 2009.

[12] L. Wang , Syst. Eng.‐Theory Pract. 2014, 34, 3079.

[13] S. Li , R.‐x. Qiu , C. Ling , Geogr. Geo‐Inf. Sci. 2008, 24, 102.

[14] H. Choi , H. Varian , Econ. Record 2012, 88, 2.

[15] C. Zhang , B.‐f. Lv , G. Peng , Y. Liu , J. Manage. Sci. China 2012, 15, 49.

[16] J.‐n. Li , Value Eng. 2013, 32, 172.

[17] J.‐F. Guo , Q. Ji , Appl. Energy 2013, 112, 1536.

[18] X. Li , J. Ma , S. Wang , X. Zhang , Econ. Model. 2015, 49, 162.

[19] M. Afkhami , L. Cormack , H. Ghoddusi , Energy Econ. 2017, 67, 17.

[20] I. Campos , G. Cortazar , T. Reyes , Energy Econ. 2017, 66, 194.

[21] J. Bai , P. Perron , J. Appl. Econ. 2003, 18, 1.

[22] A. C. Mackinlay , Econ. Lit. 1997, 13, 35.

[23] U.S. Energy Information Administration , https://www.eia.gov/ (accessed: May 2019).

[24] Google trend , https://www.google.cn/trends/ (accessed: May 2019).

